# Visual impairment and psychological distress among adults attending the University of Gondar tertiary eye care and training center, Northwest Ethiopia: A comparative cross-sectional study

**DOI:** 10.1371/journal.pone.0264113

**Published:** 2022-02-17

**Authors:** Minychil Bantihun Munaw, Melkamu Temeselew Tegegn

**Affiliations:** Department of Optometry, School of Medicine, College of Medicine and Health Sciences University of Gondar, Gondar, Ethiopia; China University of Mining and Technology, CHINA

## Abstract

**Background:**

Visual impairment can severely affect the quality of life, with a tremendous negative impact on job performance and socioeconomic status. This substantially affects the psychological status of people with visual impairment.

**Objective:**

This study aimed to determine the prevalence and factors associated with psychological distress among visually impaired Ethiopian adults attending the University of Gondar Tertiary Eye Care and Training Center.

**Methods:**

A comparative cross-sectional study was conducted at the University of Gondar Tertiary Eye Care and Training Center in Gondar City, northwest Ethiopia. A total of 206 adults with visual impairment (a presenting VA ≤ 6/18 in at least one eye) and 206 adults with normal vision were included in the study. Psychological distress was measured using a standardized self-reporting questionnaire (SRQ-20). Chi-square and binary logistic regression analysis were performed. Variables with a P-value of less than 0.05 were considered statistically significant.

**Results:**

The overall prevalence of psychological distress in the study participants was 31.07% (95% CI: 26.2, 35.8). Psychological distress was higher (43.2%; 95% CI: 36.5, 50.5) and statistically differed (p = 0.02) in visually impaired compared to adults with normal vision (18.9%; 95% CI: 14.1, 24.3). Duration of vision loss ≥ 2 years (AOR = 8.70; 95% CI: 2.38, 31.46), sudden loss of vision (AOR = 3.50; 95% CI: 1.10, 18.30), unmarried (AOR = 5.53; 95% CI: 1.66, 18.43), living lonely (AOR = 8.40; 95% CI:1.48, 4.74), College and above educational status (AOR = 2.50; 95% CI:1.47, 10.61), and loss of vision in both eyes (AOR = 1.70; 95% CI: 2.00, 14.10) were variables with a significant association with psychological distress among visually impaired adults.

**Conclusion:**

This study revealed that the prevalence of psychological distress was significantly higher among visually impaired adults than among adults with normal vision. The effect of visual impairment on psychological distress was significantly related to marital status, living arrangements, educational status, duration of vision loss, pattern of vision loss, and laterality of vision loss.

## Introduction

Visual impairment is the functional limitation of an eye or the visual system and is characterized by a decreased ability to see an object clearly, either at a distance or near, and not corrected through conventional treatments such as optical, medical, and surgical methods [1, 2]. The global prevalence of visual impairment which estimated in 2010 indicates that 285 million (4.2%) people have visual impairments, of whom 39 million (0.6%) people are blind, and 246 million (3.7%) people have low vision, and in the region of Africa, 26.3 million people have a visual impairment. Of these, 20.4 million have low vision and 5.9 million are blind. This estimation showed that 15.3% of the world’s blind population live in Africa [3]. Moreover, according to the 2017 study report, about 1.3 billion people are classified with near or distance visual impairment on a global basis [4, 5].

Visual impairment can cause physical, economic, and psychological impacts which leads to a reduced quality of life. In another way, visually impaired people have difficulty and discomfort in doing their daily living activities [1]. Furthermore, a systematic review performed by Demmin and Silverstein [6] indicates that visual impairment has a substantial personal and global impact. Their finding revealed that the rates of depression and anxiety are increasing among people with visual impairments. Besides, individuals of lower socioeconomic status may be at increased risk for developing visual impairment and subsequent mental health problems.

Several shreds of evidence indicate that the mental health status of visually impaired people is not normal [7]. Mental health is a dynamic state of internal equilibrium which enables individuals to use their abilities in harmony with the universal values of society. Basic cognitive and social skills and harmonious relationship between body and mind represent important components of mental health that contribute, to varying degrees, to the state of internal equilibrium [8]. About one-third of people with visual impairment experience subthreshold depression and anxiety, while around 5% to 7% have a major depressive disorder and 7% have an anxiety disorder, with a higher percentage than those having a healthy vision [9]. Impaired vision is associated with mental fatigue [10], less social contact and can induce feelings of loneliness and social isolation [11, 12]. Moreover, a study in Ethiopia stated that the prevalence of psychological distress is 49.6% in visually impaired adults attending Jimma tertiary eye care centre [13].

Even though visual impairment has multiple impacts in different aspects of the life of an individual like physical, socioeconomic and mental health, recent scientific evidence about this clinical morbidity on mental health is limited in Ethiopia, especially in the study area. Specifically, there is no recent study, which addresses the current epidemiologic condition of psychological distress and associated factors among visually impaired adults in Northwest Ethiopia. Therefore, this research aimed to determine the prevalence and associated factors of psychological distress in visually impaired Ethiopian adults attending the University of Gondar tertiary eye care and training center to identify and fill the gap in terms of resources and clinical decision-making which will improve early diagnosis and management of the problem.

## Methods and materials

### Study design, setting, and population

A comparative cross-sectional study was conducted at the University of Gondar comprehensive specialized hospital, tertiary eye care and training Center, Northwest Ethiopia in 2021. A consecutive sample of patients older than 17 years of age with a presenting distance Snellen visual acuity of ≤ 6/18 at least in one eye as cases, and a presenting distance Snellen visual acuity better than 6/18 in the poor eye as controls among patients attending University of Gondar tertiary eye care and training center during the study period were recruited. Patients who had severe cognitive or psychiatric conditions that could interfere with communication were excluded.

The sample size was calculated using EPI info version 7 software with an assumption of 80% of power, 95% of confidence level, exposed (persons with visual impairment) to an unexposed (person with normal vision) ratio of 1:1, a risk ratio of 2, and the prevalence of psychological distress in normal vision individuals (18.3%) [13]. The computer-generated sample size was 206 for each group.

### Data collection tool and procedure

Data was collected through face-to-face interviews, using a pre-tested structured questionnaire, and performing a physical eye examination. The interviews had two sections: socio-demographic and economic characteristics of the study participants, and questions used to measure psychological distress. The psychological distress was measured using a standardized World Health Organization self-reporting questionnaire (SRQ-20) [14]. The SRQ-20 has been adapted and validated in the Ethiopian setting to measure psychological distress. The SRQ-20 has 20 questions, asking whether the person had experienced specific symptoms over the previous one-month period. We modified the SRQ-20 to SRQ-19 based on the Ethiopian context. Those patients who gave 10 or more positive responses of SRQ-19 were considered as having psychological distress [14, 15]. The visual acuity of the study participants was measured using a Snellen distance visual acuity chart within optimal illumination, and the visual impairment was defined and categorized according to the World Health Organization definitions of visual impairment [15]. Both anterior and posterior eye examinations were performed using slit-lamp bio-microscope and 90 diopters of Volk lens to determine the causes of visual impairment.

### Independent variables

Variables included in the analysis were age, sex, marital status, religion, educational status, occupation, family monthly income, residency, family size, living arrangement, systemic co-morbid medical illness, level of visual acuity in at least one eye (mild≤6/18, moderate < 6/18-6/60, severe < 6/60-3/60, very severe <3/60-NLP), laterality of visual loss, pattern of visual loss, cause of visual loss, and duration of visual loss.

### Data analysis

After checking the completeness and consistency of the data; it was coded and entered into EPI info version 7, and exported into Statistical Package for Social Science (SPSS) version 20 software for analysis. Chi-square was done to show the statistical significance of psychological distress in visually impaired participants over participants with normal vision. Besides, binary logistic regression was fitted to identify factors associated with psychological distress among visually impaired participants. The strength of association was expressed by using an adjusted odds ratio at a 95% confidence interval. Model fitness was assured using Hosmer and Lemeshow goodness of fit. A variable with a P-value of less than 0.05 was considered statistically significant. Finally, the analyzed data was organized and presented with tables and text form as necessary.

### Ethical consideration and consent to participate

Ethical clearance was obtained from the Institutional Review Board (IRB) of the University of Gondar, College of Medicine and Health Sciences. Furthermore, an administrative written permission letter was obtained from the clinical directorate of eye care service. All the study participants were informed about the purpose of the study, their right to refuse and withdraw from the study at any time, and then verbal informed consent was obtained from each study participant. Confidentiality was also maintained by avoiding any personal identifiers from the data collection tool and using codes.

## Results

### Socio-demographic characteristics of the study participants

Out of 412 study participants, 206 were visually impaired, and 206 were normal vision adults. The majority of the study participants were living with family members (88.8%), Orthodox Christian (87.6%), and without systemic co-morbidity (87.1). Study participants in the two groups were statistical differed in terms of educational status (p = 0.00), occupation (p = 0.00), income (p = 0.00), systemic co-morbidity (p = 0.00) and psychological distress (p = 0.02). However, they were not different across religion (p = 0.93), age (p = 0.92), family size (p = 0.21) and sex (p = 0.74) (Table 1).

**Table 1:** Scio-demographic characteristics of study participants attending the University of Gondar tertiary eye care and training center, Northwest Ethiopia (n = 412).

Variables	Visual impairment	Normal vision	Total	X^2^	P-value
	No (%)	No (%)	No (%)		
**Age (in year)**				8.00	0.92
18–45	57 (27.7)	52 (25.2)	109 (26.5)		
46–56	51 (24.8)	51 (24.8)	102 (24.7)		
57–65	57 (27.7)	58 (28.2)	115 (27.9)		
66+	41 (19.6)	45 (21.8)	86 (20.9)		
**Sex**				0.10	0.74
Male	117 (56.8)	120 (58.3)	237 (57.5)		
Female	89 (43.2)	86 (41.7)	175 (42.5)		
**Marital status**				1.06	0.30
Married	137 (66.5)	109 (52.9)	246 (59.7)		
Unmarried	69 (33.5)	97 (41.1)	166 (40.3)		
**Living arrangement**				3.23	0.07
With family member	185 (89.8)	181 (87.9)	366 (88.8)		
Alone	21 (10.2)	25 (12.1)	46 (11.2)		
**Religion**				0.01	0.93
Orthodox	172 (83.5)	189 (91.7)	361 (87.6)		
Muslim	34 (16.5)	17 (8.3)	51 (12.4)		
**Educational status**				42.21	0.00
Can not read and write	100 (48.5)	25 (12.1)	125 (30.4)		
Able to read and write	47 (22.8)	59 (28.7)	106 (25.7)		
Secondary school	33 (16.1)	42 (20.4)	75 (18.2)		
College and above	26 (12.6)	80 (38.8)	106 (25.7)		
**Occupation**				34.36	0.00
Farmer	90 (43.7)	39 (18.9)	129 (31.3)		
Housewife	39 (18.9)	18 (8.7)	57 (13.8)		
Employed	33 (16)	79 (38.3)	112 (27.2)		
Unemployed	44 (21.4)	70 (34.1)	114 (27.7)		
**Income in Ethiopian Birr**				22.11	0.00
≤100	77 (37.4)	30 (14.6)	107 (26)		
1001–1500	29 (14)	29 (14.1)	58 (14.1)		
1501–3000	63 (30.6)	49 (23.8)	112 (27.2)		
>3000	37 (18)	98 (47.5)	135 (32.7)		
**Residency**				3.16	0.08
Urban	83 (40.3)	150 (72.8)	233 (56.6)		
Rural	123 (59.9)	56 (27.2)	179 (43.4)		
**Family size**				1.58	0.21
<5	97 (47.1)	103 (50)	200 (48.5)		
5+	109 (52.9)	103 (50)	212 (51.5)		
**Systemic co-morbidity**				206	0.00
No	178 (84.6)	181 (87.9)	359 (87.1)		
Yes	28 (15.4)	25 (12.1)	53 (12.9)		
**SRQ score**				4.88	0.02
<10	117(56.8)	167 (81.1)	284 (68.9)		
≥10	89 (43.2)	39 (18.9)	128 (31.1)		

### Prevalence of psychological distress

The overall prevalence of psychological distress in this study was 31.07% (95% CI: 26.2, 35.8%). The prevalence of psychological distress was higher among visually impaired participants (43.2%; 95% CI: 36.5, 50.5) than those participants with normal vision (18.9%; 95% CI: 14.1, 24.3) (Table 1).

### Factors associated with psychological distress among visually impaired patients

After conducting bivariable binary logistic regression analysis and multicollinearity diagnosis test, age, sex, marital status, educational status, laterality of vision loss, pattern of vision loss, level of vision loss, causes of vision loss, living arrangement, religion, duration of vision loss, family size and monthly income were entered into a multivariable binary logistic regression analysis. In a multivariable binary logistic regression, unmarried, living alone, college and above educational status, duration of vision loss ≥ 2 years, sudden loss of vision, and loss of vision in both eyes were remained and significantly associated with psychological distress among visually impaired study participants.

Participants with unmarried marital status were 5.53 times more likely to have psychological distress than those participants who were married (AOR = 5.53; 95% CI: 1.66, 18.43). The odds of psychological distress for participants who live alone was 8.4 times more than those participants living with family members (AOR = 8.40; 95% CI: 1.48, 4.74). Participants having college and above educational status were 2.50 times more likely to develop psychological distress than those who were unable to read and write (AOR = 2.50; 95% CI: 1.47,10.61).

The odds of psychological distress in participants with a duration of vision loss ≥ 2 years was 8.70 times more than those with a duration of vision loss < 2 years (AOR = 8.70; 95% CI: 2.38, 31.46). The occurrence of psychological distress among participants who faced sudden vision loss was 3.50 times more than participants with progressive loss of vision (AOR = 3.50; 95% CI: 1.10,18.30). The odds of psychological distress for participants with bilateral vision loss was 1.70 times more than participants with vision loss in one eye (AOR = 1.70; 95% CI: 2.00, 14.10) (Table 2).

**Table 2:** Factors associated with psychological distress among visually impaired adults attending the University of Gondar tertiary eye care and training center, Gondar city, Northwest Ethiopia.

Variables	Psychological distress (95%CI)		COR(95%CI)	AOR
	Yes(%)	No(%)		
**Age (year)**				
18–45	36 (40.4)	20 (17.1)	6.20 (2.60,14.78)	4.45(0.79,24.90)
46–56	22 (24.7)	25 (21.4)	3.04 (1.26,7.35)	4.5(0.50,8.02)
57–65	20 (22.5)	34 (29.1)	2.03(0.85,4.85)	8.4(0.58,4.42)
66+	11 (12.4)	38 (32.4)	1.00	1.00
**Sex**				
Male	48 (53.9)	69(59)	1.00	1.00
Female	41 (46.1)	48(41)	1.23 (0.70,2.14)	0.93 (0.29,2.89)
**Marital status**				
Married	46 (51.7)	91 (77.8)	1.00	1.00
Unmarried	43 (48.3)	26 (22.2)	3.27(1.79,5.98)	**5.53(1.66,18.43)** *
**Living arrangement**				
With family member	76 (85.4)	109(93.2)	1.00	1.00
Alone	13 (14.6)	8 (6.8)	2.33 (0.92,5.89)	**8.40 (1.48,4.74)** *
**Religion**				
Orthodox	68 (76.4)	104(88.9)	1.00	1.00
Muslim	21 (23.6)	13 (11.1)	2.47(1.16,5.26)	4.5(0.76,26.7)
**Educational status**				
Can not read and write	33 (37.1)	67 (57.3)	1.00	1.00
Able to read and write	19 (31.4)	28 (23.9)	1.37 (0.67,2.82)	2.33 (0.58,9.40)
Secondary school	21 (23.6)	12 (10.3)	3.55 (1.56,8.08)	5.50 (0.98,30.7)
College and above	16(17.9)	10 (8.5)	3.25 (1.33,7.94)	**2.50(1.47,10.61)** **
**Occupation**				
Farmer	27(30.3)	63 (53.8)	1.00	
Housewife	17 (19.1)	22 (18.8)	1.80(0.83,3.92)	
Employed	16(18)	17 (14.4))	2.19 (0.96,4.97)	
Unemployed	29 (32.6)	15 (12.8)	4.50 (2.09,9.74)	
**Monthly income in Ethiopian Birr**				
<1000	38 (42.7)	39 (33.3)	0.27 (0.98,0.73)	3.6(5.70,22.80)
1001–1500	6 (6.7)	23 (19.7)	1.06 (0.54,2.06)	1.03(0.17,6.24)
1501–3000	32(36)	31 (26.5)	0.55 (0.247,1.25)	2.20(0.67,13.05)
>3000	13 (14.6)	24 (20.5)	1.00	1.00
**Residency**				
Urban	45 (50.6)	38 (32.5)	2.13 (1.21,3.75)	
Rural	44 (49.4)	79 (67.5)	1.00	
**Family size**				
<5	48 (53.9)	49 (41.9)	1.00	1.00
5+	41 (46.1)	68 (58.1)	0.62 (0.35,1.07)	0.11 (0.02,5.18)
Duration of vision loss				
< 2years	28 (31.5)	47 (40.2)	1.00	1.00
2+ years	61 (68.5)	70 (59.8)	1.46 (0.82,2.61)	8.70(2.38,31.46)*
Pattern of vision loss				
Progressive	75 (84.3)	111(94.9)	1.00	1.00
Sudden	14 (15.7)	6 (5.1)	3.45 (1.27,9.39)	3.50(1.10,18.30)*
Laterality				
One eye	26 (29.2)	6 (5.1)	1.00	1.00
Both eyes	63(70.8)	111(94.9)	0.13 (0.51,0.34)	
Systemic co-morbidities				
No	77(86.5)	104(88.9)	1.00	1.70(2.00,14.10)*
Yes	12 (13.5)	13 (11.1)	1.25 (0.54,2.88)	
Causes of visual impairment				
Cataract	24(27)	55(47)	0.29 (0.15,0.59)	2.47 (0.54,11.30)
Refractive error	16(18)	23 (19.7)	0.48 (0.21,1.07)	0.35 (0.06,1.89)
Glaucoma	11 (12.4)	13 (11.1)	0.58 (0.23,1.49)	5.80(0.18,11.53)
Corneal opacity	38 (42.6)	26 (22.2)	1.00	1.00
Degree of visual impairment				
Mild	20 (22.5)	14(12)	1.00	1.00
Moderate	33 (37.1)	33 (28.2)	0.70 (0.30,1.62)	1.38 (0.03,7.05)
Severe	6 (6.7)	9 (7.7)	0.48 (0.14,1.61)	3.50(0.26,38.67)
Very severe	30 (33.7)	61 (52.1)	0.34 (0.15,0.78)	3.70 (0.06,2.17)

COR = Crude Odds Ratio, AOR = Adjusted Odds Ratio

* = p-value<0.05and

** = p-value <0.01.

## Discussion

This study tried to assess the prevalence of psychological distress in visually impaired patients attending the University of Gondar comprehensive specialized hospital tertiary eye care and training center, Gondar city, Northwest Ethiopia using a comparative cross-sectional study design. The result of this study showed that psychological distress was statistically differed among visually impaired study subjects compared to the normal vision group (p = 0.02).

In this study, the over all prevalence of psychological distress was 31.07% (95% CI: 26.2, 35.8), which was similar to previous studies done in Ethiopia [13, 16]. This might be due to nearly similar sample size was used as a study population. Besides, limited psychiatric health services in these studies areas and at the country level might be the possible reason for the similarity.

In the current study, the prevalence of psychological distress in visually impaired subjects was higher (43.2%) than that in normal vision (18.9%) adults. This is similar to meta-analysis [17], UK [18], Dutch [19], and Ethiopian Jimma [13] studies. Reduced mobility, poor communication and social engagement, and loss of independence might be the causes of a high prevalence of psychological distress in visually impaired study participants. Furthermore, this result was also higher than studies conducted in Scotland [20] and the United Kingdom [21, 22]. The possible justification for this discrepancy could be; the psychiatric health service in Scotland and United Kingdom is much better than in Ethiopia.

However, the result of this study was lower than a meta-analysis study [23], and studies conducted in the United States of America [24], United Kingdom [25, 26], and Nigerian [27] studies. The possible difference might be the study setting. The above studies were conducted at the community level, but our study was conducted at a hospital, which might underestimate the prevalence of psychological distress.

Visually impaired participants currently unmarried were five times more likely to develop psychological distress as compared to married adults. individuals with visual impairment have difficulty of doing activities of daily living, which leads to dependency. On the other hand, those individuals have not sufficient support in different affairs can cause loneliness. These increase depression and anxiety among visually impaired participants.

Visually impaired study participants with college and above educational status were two times more likely to develop psychological distress as compared to those who can not read and write. This result is similar to the study conducted in Nigerian [28]. This might be due to loss of job and reduced social participation as a result of reduced visual function.

The likelihood of developing psychological distress in visually impaired participants who live lonely was eight times higher than those who live with family members (wife, husband, child, mother, and father). This finding is supported by a study done in Mekelle, Ethiopia [16].The possible explanation for this could be individuals living alone have reduced social support, which can cause isolation. This is more aggravated in those individuals with visual impairment.

Visually impaired study participants with a duration of vision loss two years and above in either eye had almost nine times higher risk of developing psychological distress relative to participants with a duration of vision loss less than 2years. The description could be as the duration of vision loss increases, patients will lose their hope for visual recovery, which leads to depression and anxiety.

The likelihood to develop psychological distress in visually impaired study participants with a sudden loss of vision in either eye was three times higher than those participants with progressive loss of vision. Sudden loss of vision can lead to unfortunate socio-economic disturbance like losing financial leadership and source of income. Usually, this occurs in an unexpected manner that might put the person under stress.

Study participants with bilateral visual impairment were two times more likely to develop psychological distress as compared to those participants with monocular visual impairment. This finding is in line with a study conducted in Jimma, Ethiopia [13]. This indicates that psychological distress was significantly associated with the laterality of visual impairment. It describes people with bilateral loss of vision are more prone to develop psychosocial distress than people with loss of vision in one eye because those individuals are more compromised in making money through a job, social engagement, and doing activities of daily living.

Although this study was the only study in Northwest Ethiopia that describes the association between psychological distress and visual impairment, it had different limitations. Since adults with cognitive impairment and severe mental illness that interferes in communication during data collection were excluded, this underestimates the prevalence of psychological distress. Moreover, the instrument used was basically helps to identify general psychological distress, not specific mental illnesses.

In conclusion, the prevalence of psychological distress was significantly higher among visually impaired as compared to normal vision adults. The effect of visual impairment on psychological distress was significantly related to marital status, living arrangement, educational status, duration of vision loss, pattern of vision loss, and laterality of vision loss. Therefore, to overcome the impact of visual impairment on Psychological status, it is important to integrate psychological care into the current eye care service in medical and surgical units.

## Supporting information

S1 File (DOCX)

S2 File (SAV)

S3 File (SAV)
