# Supplementary material for: Visual impairment and psychological distress among adults attending the University of Gondar tertiary eye care and training center, Northwest Ethiopia: A comparative cross-sectional study
Source: PLoS One. 2022 Feb 17;17(2):e0264113. doi: 10.1371/journal.pone.0264113 (PMC8853488; doi:10.1371/journal.pone.0264113)
Supplement: S1 File — (DOCX) [file pone.0264113.s001.docx]

Data extraction tool for visual impairment and psychological distress among visually impaired patients- comparative cross sectional study

የስምምነት ማስሞያ ቅጽ

ስሜ ____________________ይባላል፡፡ በአሁኑ ወቅት በጎንደር ዩኒቨርሲቲ እየሰራለሁ እገኛለሁ፡፡

የጥናቱ ዋና አላማ በዓይን ዕይታ ችግር ምክንያት የሚፈጠር የአእምሮ ጤና ችግርን ለማጥናት ነው፡፡ ጥናቱ ካለው ፋይዳ አንፃር እርስዎ የዚህ ጥናት ተሳታፊ ይሆኑ ዘንድ ተመርጠዋል፡፡ ይህም በችግሮቹ ዙሪያ አስፈላጊውን እርምጃ ለመውሰድ እና የተሻለ የአይን ጤና አገልግሎት እንዲኖር ያደርጋል፡፡ መጠይቁ ጥናቱን ለማከናዎን ጠቃሚ ስለሆነ እንዲሞሉ እየተጠየቁ ስምዎ በመጠይቁ አይጻፍም፤ የሚሰጡትም መልስ ሙሉ በሙሉ ምስጢራዊነቱ ተጠብቆ ለጥናቱ ብቻ የሚውል ነው፡፡ ስለዚህ በሂደቱ ላይ እንደአስፈላጊነቱ በነጻነት ይሙሉ ምንም ሊያስፈራዎት አይገባም፤ ከዚህ በላይ ደግሞ እርስዎ መሙላት የማይፈልጉት ጥያቄ ቢኖር ለመሙላት አይገደዱም፡፡ ለጥያቄው ከ20-30 ደቂቃ በቂ ሲሆን ምንም አይነት አስተያየት እና ጥያቄ ቢኖረዎት ከዚህ በታች የተሰጠውን አድራሻ በመጠቀም ሊገናኙን ይችላሉ፡፡

በጥናቱ ለመሳተፍ ፈቃደኛ ነዎት ? አዎን አይደለሁም

አመሰግናለሁ፡፡

የጥናቱን አላማና ጥቅም ተገንዝቤ በዚህ ጥናት ለመሳተፍ ወስኛለሁ::

ፊርማ ___________________________________ ቀን _____________________________

ቃለ-መቀይቅ አቅራቢው

ስም_______________________________________________ፊርማ ____________ ቀን __________

አድራሻ፡- ስም፡- ምንይችል ባንተይሁን

ስልክ ቁጥር 251-918-28-23-16

Email: minychilmedban@gmail.com

**ክፍል I: ማህበራዊ መረጃ**

1. እድሜ በ ዓመት-----------------------
2. ፆታ ሀ. ወንድ ለ. ሴት
3. የጋብቻ ሁኔታ

ሀ. ያላገባ/ች ለ. ያገባ/ች ሐ. የፈታ/ች መ. የሞተበት/ባት

1. የቤተሰብ ቁጥር_____________________________________
2. የትምህርት ደረጃ

ሀ. ማንበብ እና መፃፍ የማይችል/ትችል ሐ. የመጀመሪያ ደርጃ ሰ. ኮሌጅ እና ከዚያ በላይ

ለ. ማንበብ እና መፃፍ የሚችል/የምትችል መ. የሁለተኛ ደረጃ

1. የመኖሪያ ቦታ ሀ. ከተማ ለ. ገጠር
2. ስራ

ሀ. ገበሬ ለ. የቀን ሰራተኛ ሐ. ባለሙያ/ ቅጥረኛ መ. ጥሮታ የወጣ/ች ሰ. ስራ አጥ

ረ. ተማሪ ሠ. የቤት እመቤት ሸ. ነጋዴ

1. ሐይማኖት

ሀ. ኦርቶዶክስ ለ. ሙስሊም ሐ. ፕሮቴስታንት መ. ካቶሊክ ሰ. ሌላ ካለ ይጥቀሱ……………

1. የሚኖሩት ከ ማን ጋር ነው

ሀ. ከቤተሰብ ጋር (እናት፡ አባት፡ እህት፡ ወንድም፡ ባል፡ ሚስት) ለ. ለብቻ

ሐ. ከዘመድ ጋር መ. ሌላ ካለ ይጥቀሱ----------

1. በቤተሰብ ውስጥ የእይታ ችግር ያለበት ሰው አለ

ሀ. አዎ ለ. የለም ሐ. አላውቅም

1. የቤተሰብ ጠቅላላ ወርሃዊ ገቢ በ ኢትዮጵያ ብር ----------------------

ክፍል II: የአእምሮ ጤናን መፈተሻ ጥያቄዎች(አልፎ አልፎ ለሚሉ ሰዎች አዎ እንደማለት ይቆጠራል)

1. አብዛኛውን ጊዜ ራስ ምታት ያመዎታል? ሀ. አዎ ለ. አያመኝም
2. የምግብ ፍላጎትዎ የቀነሰ ነው? ሀ. አዎ ለ. አይደለም
3. የእንቅልፍ ችግር አለብዎት? ሀ. አዎ ለ. የለብኝም
4. በአዲስ አካባቢ በሚሆኑበት ጊዜ ይፈራሉ? ሀ. አዎ ለ. አልፈራም
5. የእጅ መንቀጥቀጥ ችግር አለብዎት? ሀ. አዎ ለ. የለብኝም
6. የመፍራት፣ የመወጠር ወይም የመጨነቅ ስሜት ይሰማዎታለ? ሀ. አዎ ለ. አይሰማኝም
7. ምግብ አልፈጭ ይልዎታለ? ሀ. አዎ ለ. አይለኝም
8. የማስታወስ ወይም የማሰላሰል ችግር አለብዎት? ሀ. አዎ ለ. የለብኝም
9. አብዛኛውን ጊዜ ይከፋሉ? ሀ. አዎ ለ. አልከፋም
10. አብዝተው የማልቀስ ነገር አለ? ሀ. አዎ ለ. አላለቅስም
11. የቀን ተቀን ስራዎን ለማከናዎን ይቸገራሉ? ሀ. አዎ ለ. አልቸገርም
12. በቀን ተቀን ውሎዎ ውሳኔ ለመዎሰን ይቸገራሉ? ሀ. አዎ ለ. አልቸገርም
13. የቀን ተቀን ስራዎ ስቃይ ይሆንብዎታል? ሀ. አዎ ለ. አይሆንብኝም
14. በራስዎት ምክንያት በመማህበረሰቡ ውስጥ ያለዎት ተሳተፎ ዝቅተኛ ነው? ሀ. አዎ ለ. አይደለም
15. ለሚሰሩት ስራ ፍላጎት የማጣት ስሜት አለዎት? ሀ. አዎ ለ. የለብኝም
16. ለራስዎት የሚሰጡት ዋጋ ዝቅተኛ ነው? ሀ. አዎ ለ. አደለም
17. በራስዎት ላይ የግድያ እርምጃ (ራስን ማጥፋት) ለመውሰድ አስበው ያውቃሉ? ሀ. አዎ ለ. አላውቅም
18. አብዛኛውን ጊዜ የድካም ስሜት ይሰማዎታለ? ሀ. አዎ ለ. አይሰማኝም
19. ሆድዎትን ያለመመቸት ስሜት ይሰማዎታለ? ሀ. አዎ ለ. አይሰማኝም
20. ከእንቅልፍዎት እንደነቁ የድካም ስሜት ይሰማዎታል? ሀ. አዎ ለ. አይሰማኝም

ክፍል III: Visual status and related questions

1. Distance visual acuity

1. Unaided visual acuity, A. OD----------- B. OS-------------- C. OU_______

2. with current spectacle, A. OD_______ B. OS_________ C. OU_______

**If the participant has visual reduction less or equal to 6/12 in either eye continue to fill the following**.

1. Pattern of vision loss

**OD** A. Progressive B. Sudden **OS**  A. Progressive B. Sudden

36. Causes of vision loss/ diagnosis (more than one choice is possible)

OD A. Cataract B. Glaucoma C. Refractive error D. Corneal opacity

E. Uveitis F. AMD G. Retinal detachment J. others list----------

OS A. Cataract B. Glaucoma C. Refractive error D. Corneal opacity

E. Uveitis F. AMD G. Retinal detachment J. others list---------

1. Duration of vision loss

OD__________________ OS___________________

40. The eye having visual impairment

A. OD B. OS C. OU

41. Systemic disease__________________________________, ­­­___________________
